# Absorption of* Codonopsis pilosula* Saponins by Coexisting Polysaccharides Alleviates Gut Microbial Dysbiosis with Dextran Sulfate Sodium-Induced Colitis in Model Mice

**DOI:** 10.1155/2018/1781036

**Published:** 2018-08-19

**Authors:** Yaping Jing, Anping Li, Zhirong Liu, Pingrong Yang, Junshu Wei, Xinjun Chen, Tang Zhao, Yanrui Bai, Lajia Zha, Chunjiang Zhang

**Affiliations:** ^1^School of Life Sciences, Lanzhou University, Lanzhou 730000, China; ^2^Key Laboratory of Cell Activities and Stress Adaptations, Ministry of Education, Lanzhou University, Lanzhou 730000, China; ^3^Gansu Key Laboratory of Biomonitoring and Bioremediation for Environmental Pollution, Lanzhou University, Lanzhou 730000, China; ^4^Gansu Institute of Drug Control, Lanzhou 730030, China; ^5^Gansu Key Laboratory of Functional Genomics and Molecular Diagnosis, Lanzhou 730000, China

## Abstract

**Objectives:**

Inflammatory Bowel Disease (IBD) is an autoimmune disease, and the gut microbiota has become a new therapeutic target. Herbal medicine (HM) has shown good efficacy in the clinical treatment of IBD; however, the synergistic actions of the dominant chemicals in HM decoctions are unclear.

**Methods:**

In this study, we explored whether the complicated interconnections between HM and the gut microbiota could allow crosstalk between HM ingredients. Saponins and polysaccharides, i.e., the dominant chemicals in the* Codonopsis pilosula* Nannf (CPN) decoction, were investigated in a dextran sulfate sodium- (DSS-) induced mouse model. Bacterial 16S rRNA sequencing analyzed the change of gut microbiota structure and diversity. Gas chromatography (GC) determined the content of short-chain fatty acids (SCFAs) in feces. ELISA detected the expression of proinflammatory and anti-inflammatory cytokines associated with TH17/Treg balance. UPLC-QTOF-MS technology combined with PKsolver software analyzed the absorption of the highest exposure for monomeric compounds of CPN saponins in serum. The results indicated that CPN polysaccharides showed prebiotic-like effects in mice with DSS-induced colitis by simultaneously stimulating the growth of three important probiotics, i.e.,* Bifidobacterium* spp.,* Lactobacillus* spp., and* Akkermansia* spp., and inhibiting the growth of pathogenic bacteria, including* Desulfovibrio* spp.,* Alistipes* spp., and* Helicobacter* spp. Moreover, CPN polysaccharides improved intestinal metabolism, enhanced the production of short-chain fatty acids, upregulated the expression of anti-inflammatory cytokines and downregulated the secretion of proinflammatory cytokines correlated with Th17/Treg balance, promoted the absorption of certain CPN saponins in the serum, and stimulated recovery of the holistic gut microbiota.

**Conclusion:**

CPN polysaccharides have the good prebiotic properties and shown good application prospects in the prevention and treatment of acute colitis. These findings provide insights into the specific bacteria responsible for active, inactive biotransformation of HM ingredients and those that are altered by HM administration.

## 1. Introduction

Inflammatory bowel disease (IBD) is a chronic disorder of the lower gastrointestinal (GI) tract, encompassing two major diseases: Crohn's disease (CD) and ulcerative colitis (UC) [1]. Environmental factors, diet, susceptibility genes [2], inappropriate immune responses [3], and gut microbes are known to be involved in the pathogenesis of IBD [4]. Molodecky et al. (2012) found that the incidence of IBD was significantly associated with race and geography [5]. Within the past decade, IBD has emerged as a global public health challenge [6], with the highest incidence rates observed in developed countries, such as North America, Europe, Australia, and New Zealand [7]. Currently, IBD is also common in developing countries, such as countries in Asia and South America, including Brazil, South Korea, and China [8, 9], with high prevalence and rapidly increasing incidence rates. IBD commonly affects young people as a chronic disorder [1] and is conventionally treated with aminosalicylic acids [10, 11], corticosteroids [12], immune suppressants [13, 14], antibiotics [15], and biologic agents [16]. However, these agents are expensive and do not completely prevent colitis. Moreover, most patients eventually become immune tolerant to these drugs, and the side effects related to their use are quite extensive, with some being life-threatening [17]. Therefore, new treatments for IBD are urgently needed.

The gut microbiota has formed as a result of symbiosis between symbiotic microbes and animals over at least 500 million years of coevolution [18]. Notably, the gut microbiota is mainly composed of bacteria, fungi, archaebacteria, and viruses [19] and plays a critical role in maintaining gut homeostasis and host health [20]. IBD generally occurs in the colon, rectum, ileum, and other parts of the GI tract that come in contact with bacteria [21]. Microbiota studies have shown that the gut of patients with IBD exhibits reduced diversity compared with that of healthy controls [22, 23]. In addition, gut microbial dysbiosis, which refers to alter composition of the gut microbiota associated with functional changes in the microbial metabolome, is a major feature in IBD and is often associated with decreased probiotics and increased opportunistic pathogens in gut microbiota [24]. Synthesis of certain nutrients, particularly short-chain fatty acids (SCFAs), by gut symbiotic bacteria fermentation of undigested carbohydrates in the colonic lumen [25, 26], can improve intestinal barrier permeability and metabolism, regulate the energy balance of the host, and modulate anti-inflammatory effects [27–29] by releasing energy locally in the colon and systemically in the liver via the portal bloodstream after being absorbed by host cells [30, 31]. Moreover, gut microbial dysbiosis is linked to aberrant immune responses, which are often accompanied by abnormal production of inflammatory cytokines [32], such as the production of T-helper 17 (Th17)/regulatory T cell- (Treg-) related signature cytokines, including interleukin- (IL-) 17A, IL-21, IL-22, IL-23, and IL-25, which can promote tissue inflammation via the induction of other proinflammatory cytokines and chemokines [33]. Therefore, restoration of the healthy gut microbiome has become a new goal of IBD therapy [34].

Complementary and alternative medicine (CAM) has been accepted as a viable therapeutic approach for patients with IBD because of its efficacy and mild side effects compared with western drugs [35]. Herbal medicine (HM), the primary type of CAM, has been used for the treatment and prophylactic management of diseases for centuries [36]. Moreover, HM has the advantage of using multiple components against multiple targets, resulting in good efficacy in the treatment of various diseases [37]. Many studies have shown that the gut microbiota plays a crucial role in HM therapy. For example, the gut microbiota converts HM components into metabolites that have different bioavailabilities and bioactivities from their precursors and mediates the interactions between the multiple chemicals in HM (synergistic and antagonistic) [38]. Additionally, HM chemicals improve the composition of the gut microbiota, consequently ameliorating dysfunctions and associated pathological conditions [39]. Noncarbohydrate small molecules and polysaccharides are the dominant chemicals in HM decoctions [40]. Moreover, HM polysaccharides, even without absorption, are still capable of affecting host physiological and pathological conditions by interacting with the gut microbiota [37]. For example, the abundances of* Bifidobacterium* and* Lactobacillus* are significantly increased after administration of* Astragalus* polysaccharides in 2,4,6-trinitrobenzene sulfonic acid-induced colitis, whereas those of* Enterococcus* and* Enterobacteriaceae* are decreased [41].* Purslane* polysaccharide also enhances the growth of* Bifidobacteria* and* Lactobacillus* and reduces endotoxin content in the peripheral blood in dextran sulfate sodium- (DSS-) induced colitis [42]. In contrast, noncarbohydrate small molecules, such as saponins, are often metabolized to secondary glycosides and/or aglycones with better bioavailability and bioactivity by enzymes encoded in intestinal bacteria (frequently probiotics) [43, 44]; however, an appropriate reference substance is difficult to obtain due to the complexity of the composition. In addition, research on the separation and purification of monomer components is limited by the use of analytical chemistry to study the absorption and distribution of saponins in vivo, restricting the clinical research and development of active ingredients from HMs.

*Codonopsis pilosula* (Franch) Nannf (CPN), a classical traditional Chinese herbal formulation, is sometimes used as a substitute for Ginseng (*Panax ginseng* C. A. Mey) to widely replenish Qi (vital energy) deficiency, strengthen the immune system, improve poor GI function, alleviate gastric ulcers, and improve appetite [45, 46]. The representative chemicals of CPN, including polysaccharides and saponins, are thought to be responsible for most activities in plants of this genus [47]. Because previous studies have used total saponins but not monosodium saponins of CPN, it has been difficult to study the synergistic effects of polysaccharides and saponins in CPN on certain diseases. Based on the prebiotic effects of HM polysaccharides [48], we assumed that in the DSS-induced colitis mouse model, CPN polysaccharides, although not directly digestible, may function as prebiotics, leading to favorable changes in the gut microbiota by promoting the growth of probiotics; the improved gut microbiota may then enhance the absorption of bioactive saponins coadministered in the CPN.

Accordingly, in this study, we used the relative exposure method and dilution ratio curve [49] to study the dynamic changes in the levels of total saponins in CPN. We then examined the effects of polysaccharides on the intestinal metabolism and absorption of saponins in CPN and explored the gut microbiota-mediated mechanisms involved in DSS-induced acute colitis in mice.

## 2. Materials and Methods

### 2.1. Chemicals and Reagents

DSS (molecular weight: 36,000–50,000 Da) was obtained from Sigma-Aldrich (St. Louis, MO, USA). High-performance liquid chromatography-grade acetonitrile and methanol were purchased from Tedia Co, Inc. (Fairfield, OH, USA). Fructooligosaccharides (Fro) were provided by BENEO GmbH (Mannheim, Germany). The standards for acetate, propionate, butyrate, isobutyric, isovaleric, valeric acid, and 2-ethyl butyric acid were purchased from Shanghai Macklin Biochemical Co., Ltd. The kit for biochemical analysis of myeloperoxidase (MPO) was purchased from Jiancheng Bioengineering Institute (Nanjing, China). The mouse enzyme-linked immunosorbent assay (ELISA) kits for cytokines analysis were purchased from Dakewe (Shenzhen, China). Ultra-high-performance liquid chromatography triple quadrupole mass spectrometry (UPLC-TQ-MS) and gas chromatography (GC) instruments were purchased from Agilent Biological Company (Shanghai, China).

### 2.2. Preparation of CPN Polysaccharides and Saponins

CPN polysaccharides (56%) and saponins (95%) were purchased by the Lanzhou Waters Technology Co., Ltd. (Lanzhou, China).

### 2.3. Ethics Statement

All animal studies strictly followed institutional and international ethical guidelines for the care and use of laboratory animals. All methods were carried out in accordance with the protocols approved by Lanzhou University's Ethics Committee of Animal Experiments.

### 2.4. Animal Experimental Design

Female C57BL/6 mice (18 ± 2 g, 4 weeks old at the beginning of the trial) were purchased from the Animal Experimental Center of the Chinese Academy of Sciences. Each animal was evaluated to be in good health and then acclimated to the laboratory environment for 1 week. Mice were housed individually at 20 ± 4°C with an alternating 12 h light/dark cycle, 35–55% relative humidity, and animal feed and clean water provided ad libitum.

In this study, the entire experiment was divided into prevention period (the first day to the 21^th^ day) and treatment period of acute colitis (the 22^th^ to the 30^th^ day). Female C57BL/6 mice were randomly divided into five groups (n = 18 per group: Fro,* Codonopsis pilosula* polysaccharide [Cp],* Codonopsis pilosula* saponin [Cs], normal control [Con], and DSS-induced colitis [Mod] groups). Fecal samples were collected once from each group before the experiment began. Subsequently, the Fro, Cp, and Cs groups were treated with 300 mg/kg of different drugs once a day for 21 days (prevention period), and the Con and Mod groups were treated with an equal volume of distilled water. Finally, in addition to the Con group, all mice were given drinking water containing 3% DSS for 7 days (from the 22^th^ to 28^th^ days; the modeling period), resulting in an acute colitis model [50]. Fresh DSS solution and distilled water were prepared daily. During this period, feces were collected twice on days 21 and 30. After fasting for 12 h (day 29), all groups were administered CPN saponin solution (300 mg/kg) once (day 30), and biological samples were collected to observe the absorption of CPN saponin in serum of mice model with acute colitis after a period of different drug interventions. The experimental process for induction of murine colitis by 3% DSS is shown in Figure 2(a).

During the whole experiment period, the dosage was determined according to the pharmacopoeia regulations (which recorded the commonly used dose of CPN was 9-30g, and we took the maximum amount of 30g). Combined with the relevant literature reports [51], the final dosage of Cp and Cs is 300mg/kg by converting the ratio of body surface area of animals and humans.

At the end of the experiment, the mice were anesthetized with ether and sacrificed. Blood samples were quickly collected by the eye-catching blood method, and colons were quickly removed, opened longitudinally, and gently cleared of stool by phosphate-buffered saline (PBS). Macroscopic assessment of the disease grade was scored according to a previously reported scoring system [52] and the colon tissues were then used for immunoblotting and ELISA analysis.

### 2.5. Fecal Sample Collection

Fecal samples (approximately 0.5 g) were collected immediately after deposition and stored on ice on days 1, 21, and 30. Samples were then frozen at –80°C until 16S rRNA sequencing and SCFA analysis.

### 2.6. Blood Sample Collection

Blood samples (approximately 100 *μ*L) were collected from the retinal vein plexus into test tubes containing sodium heparin at 1, 3, 6, 9, 12, and 24 h ( n = 3 per point) after CPN saponin administration. Samples were then stored at –20°C until further analysis.

### 2.7. Histological Analysis

Hematoxylin and eosin- (H&E-) stained slides were used to evaluate histological damage to the colon. Colonic histology was scored blindly according to standard protocols [53].

### 2.8. Assay of MPO Activity

The MPO activity of colon tissue was defined using an MPO kit (Jian Cheng Bioengineering Institute) according to the manufacturer's instructions.

### 2.9. Analysis of Inflammatory Cytokines

The colon tissue was ground with a pestle in PBS (15 volumes) and then centrifuged for 15 min at 5000 ×* g*. The supernatant was collected for analysis according to the manufacturer's instructions with commercially available mouse ELISA kits (Dakewe) for the following disease-specific cytokines: IL-17A, IL-17F, IL-6, IL-22, tumor necrosis factor (TNF)-*α*, transforming growth factor (TGF)-*β*, and IL-10.

### 2.10. Determination of SCFAs in Feces

A portion of each fecal sample was thawed at room temperature, and 0.1 g samples were suspended in 0.5 mL PBS and centrifuged for 10 min at 3000 ×* g*. The supernatant (1 mL) was mixed with 0.2 mL metaphosphate-deproteinized solution (25 g metaphosphoric acid and 0.217 mL 2-ethyl butyric acid in 100 mL distilled water), and the mixture was then centrifuged at 10000 ×* g* for 12 min at 4°C after incubating on ice for 30 min. The supernatant was collected for GC analysis, which was performed on an Agilent 7890 series with a flame ionization detector (FID). The chromatographic separation was achieved with a high-efficiency capillary column (DB-FFAP; 30 m × 0.25 mm, 0.25 *μ*m). The maximum temperature was 250°C, the carrier gas was high-purity nitrogen, the FID temperature was 230°C, the inlet temperature was 250°C, and split ratio was 30: 1. The injection conditions were as follows: initial injection temperature, 60°C, maintained for 1 min; temperature increased to 220°C at 20°C/min, maintained for 3 min. The injection volume was 1 *μ*L.

### 2.11. 16S rRNA Gene Sequence Analysis in Fecal Samples

Total DNA was isolated from 60 fecal samples (four mice were randomly selected from each group) using an AccuPrep Stool DNA Extraction Kit. The V3-V4 region of the bacterial 16S ribosomal RNA gene was amplified using TransStart Fastpfu DNA Polymerase in a volume of 20 *μ*L containing 10 ng template DNA, 4 *μ*L 5× FastPfu Buffer, 2 *μ*L 2.5 mM dNTPs, 0.8 *μ*L forward primer (5 *μ*M), 0.8 *μ*L reverse primer (5 *μ*M), 0.4 *μ*L FastPfu polymerase, and ddH_2_O to 20 *μ*L. The amplification conditions were 95°C for 5 min (initial denaturation), followed by 27 cycles of denaturation at 95°C for 30 s, annealing at 55°C for 30 s, and extension at 72°C for 45 s; the sample was then incubated for 10 min at 72°C and held at 10°C. The primers used in this study were as follows: 515F (5′-GTGCCAGCMGCCGCGG-3′) and 806R (5′-GGACTACHVGGGTWTCTAAT-3′), where the barcode was a base sequence unique to each sample.

The PCR products were extracted from 2% agarose gels and purified using an AxyPrep DNA Gel Extraction Kit (Axygen Biosciences, Union City, CA, USA) according to the manufacturer's instructions and quantified using QuantiFluor-ST (Promega, WI, USA). The products were then sequenced on an Illumina MiSeq 250 sequencing platform according to the “Sequencing Methods” manual [54].

### 2.12. Analysis of the Absorption of CPN Saponins in Blood by UPLC-TQ-MS

Blood samples were thawed at room temperature and then treated as follows. First, samples were centrifuged for 15 min at 8000 ×* g*. The plasma was deprotonized, and supernatants (100 *μ*L) were mixed with 300 *μ*L methanol. The mixture was then centrifuged at 10000 ×* g* for 12 min at 4°C. The supernatant was collected for UPLC-TQ-MS analysis, which was performed on an Agilent 6460 Triple Quadrupole Mass Spectrometer with an electrospray ionization (ESI) source, coupled with an UPLC Agilent 1260 series instrument.

The chromatographic separation was achieved with an Agilent Poroshell 120 EC-C18 column (2.1 mm × 100 mm, 2.7 *μ*m). The mobile phase consisted of (A) 0.1% formic acid in water and (B) acetonitrile containing 0.1% formic acid. The elution conditions were optimized as follows: 25–65% B (0–4 min), 65–85% B (4–6 min), 85–25% B (6–6.1 min), and 25% B (6.1–9 min). The flow rate was 0.25 mL/min. The column and autosampler were maintained at 30°C. The injection volume was 2 *μ*L. Mass spectra were acquired in ESI mode using nitrogen gas at a temperature of 350°C, a nebulizer pressure of 15 psi, a flow rate of 12 L/min, and a capillary voltage of 3000 V. Total ion chromatograms were obtained from* m/z* 200 to* m/z* 1500 in MS/MS positive mode.

### 2.13. Statistical Analysis

Statistical analysis was performed using SPSS 22 software. Data were plotted in the figures as means ± standard errors of the means. Differences between two groups were analyzed using one-way analysis of variance and assessed using two-tailed tests. Gut microbial analysis performed using R language and Origin 8.0 software. Differences with* p *values of less than 0.05 were considered significant.

The pharmacokinetic parameters of CPN saponins were analyzed by a noncompartmental method using the nonlinear least squares regression program PKsolver 2.0 (Chinese Pharmaceutical University) [55].

## 3. Results

### 3.1. Component Analysis of CPN Saponins

**Figure 1:**
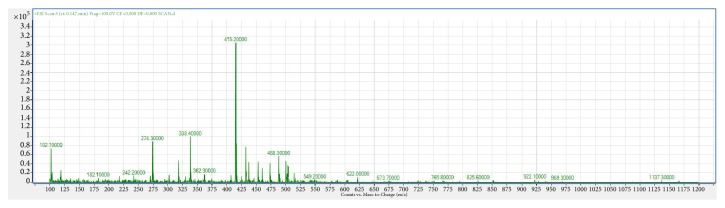
Mass spectrum of* Codonopsis pilosula* saponins in positive ESI mode.

Analysis parameters of the tranexamic acid mass spectrum are shown in Table 1. The mass spectrometry full scan of CPN extracts (Figure 1) showed that CPN saponins gave a fairly strong mass response in positive ESI mode, and the total amount of saponins with the highest exposure was predominantly deprotonated molecular ions [M–H]^–^ at* m/z* 415.2, hereinafter referred to as Cs-415.2.

### 3.2. Amelioration of Colitis with Different Drugs

**Figure 2:**
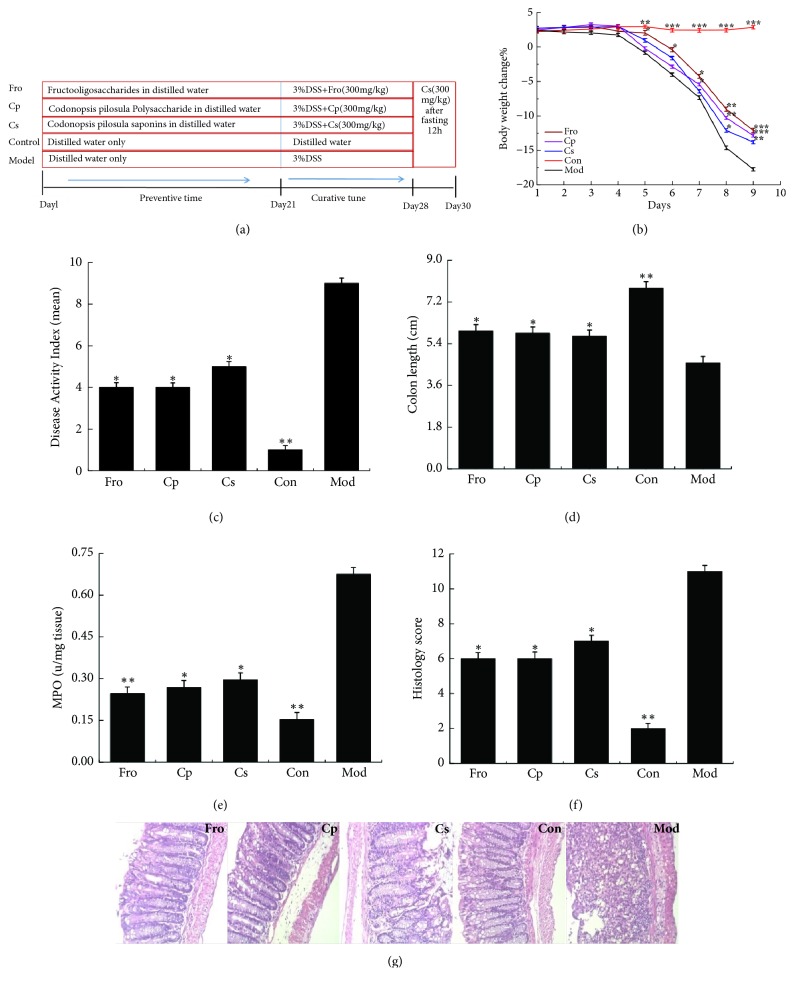
Role of* Codonopsis pilosula* extracts in the DSS-induced colitis model in C57BL/6 mice. (a) Experimental process of UC induction by 3% DSS. (b) Body weight change after induction of colitis using 3% DSS. DAI (c), colon length (d), MPO (e), and histology score (f) in each group. (g) Representative H&E-stained colorectal sections (100× magnification) in mice with acute colitis. Histology score based on 10 H&E-stained sections per mouse. ^*∗*^*P* < 0.05; ^*∗∗*^*P* < 0.01; ^*∗∗∗*^*P* < 0.001 versus the DSS-treated group. Data are presented as means ± SD of four mice in each group (b–f, n = 4). Fro = fructooligosaccharides group, Cp =* Codonopsis pilosula* polysaccharide group, Cs =* Codonopsis pilosula* saponins group, Con = control group, and Mod = DSS-induced colitis group.

The effects of CPN extracts on DSS-induced colitis in C57BL/6 mice are shown in Figure 2. During the modeling period, after colitis was induced in mice by administration of 3% DSS solution for 7 days (from the 22^th^ to 28^th^ day), the body weight of mice in the Mod group was significantly lower than that in the Con group (*p *< 0.01), whereas mice in the drug groups (Fro, Cp, and Cs) showed marked inhibition of body weight loss in the last 2 days (*p *< 0.01; Figure 2(b)). The disease activity index (DAI) score, which was used to evaluate inflammation severity in colitis, increased significantly after DSS intake (*p *< 0.01) and was markedly attenuated (*p *< 0.05) in the Fro, Cp, and Cs groups (Figure 2(c)). In the Mod group, a significant reduction of colon length was observed compared with that in the Con group (*p *< 0.01), whereas shortening of the colon was markedly reduced in the Fro, Cp, and Cs groups compared with that in the Mod group (*p *< 0.05; Figure 2(d)). MPO activity (Figure 2(e)) and histology scores (Figure 2(f)) were also increased markedly by DSS treatment compared with that in the Con group (*p *< 0.05); however, Fro, Cp, and Cs ameliorated these effects in the DSS-induced colitis model (*p *< 0.05). H&E-stained colorectal sections showed distortion of crypts, loss of goblet cells, severe epithelial injury, and inflammatory cell infiltration in the mucosa and submucosa caused by DSS treatment in the Mod group (Figure 2(g)). The drug groups exhibited different degrees of protection against histological inflammation and colon crypt structures.

### 3.3. Effects of Different Drugs on Colon Cytokine Levels Associated with the Th17/Treg Balance in Mice with DSS-Induced Colitis

**Figure 3:**
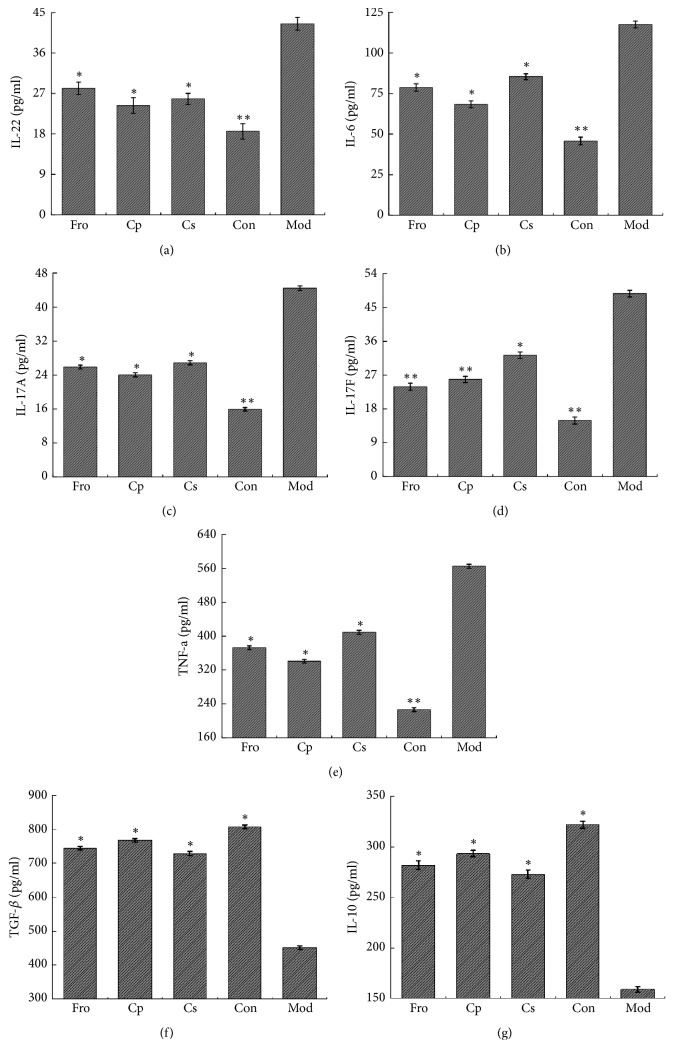
Expression of proinflammatory and anti-inflammatory cytokines associated with the Th17/Treg balance in mice with DSS-induced colitis. (a) Expression levels of IL-22, (b) IL-6, (c) IL-17A, (d) IL-17F, (e) TNF-*α*, (f) TGF-*β*, and (g) IL-10 in the colorectum, as determined by ELISA. ^*∗*^*P* < 0.05; ^*∗∗*^*P* < 0.01; ^*∗∗∗*^*P* < 0.001 versus the DSS-treated group. Data are presented as means ± SD of four mice in each group (a–g, n = 4). Fro = fructooligosaccharides group, Cp =* Codonopsis pilosula* polysaccharide group, Cs =* Codonopsis pilosula* saponins group, Con = control group, and Mod = DSS-induced colitis group.

IL-17A, IL-17F, IL-6, IL-22, and TNF-*α* are the most important proinflammatory cytokines produced by Th17 lymphocytes [56]. As shown in Figures 3(a)–3(e), the levels of IL-22, IL-6, IL-17A, IL-17F, and TNF-*α* were significantly increased in mice with DSS-induced colitis compared with those in the Con group (*p *< 0.01). However, treatment with the different drugs (Fro, Cp, and Cs) markedly inhibited the expression of IL-17A, IL-17F, IL-6, IL-22, and TNF-*α* compared with that in the Mod group. As shown in Figures 3(f) and 3(g), TGF-*β* and IL-10 levels, as the major cytokines participating in Treg immune function, were markedly reduced in mice with DSS-induced colitis compared with those in the Con group (*p *< 0.05); in contrast, mice in the Fro, Cp, and Cs group showed markedly enhance expression of TGF-*β* and IL-10 compared with mice in the DSS-induced colitis group (*p* < 0.05).

### 3.4. Overall Structural Modulation of the Gut Microbiome after Treatment with Different Drugs

**Figure 4:**
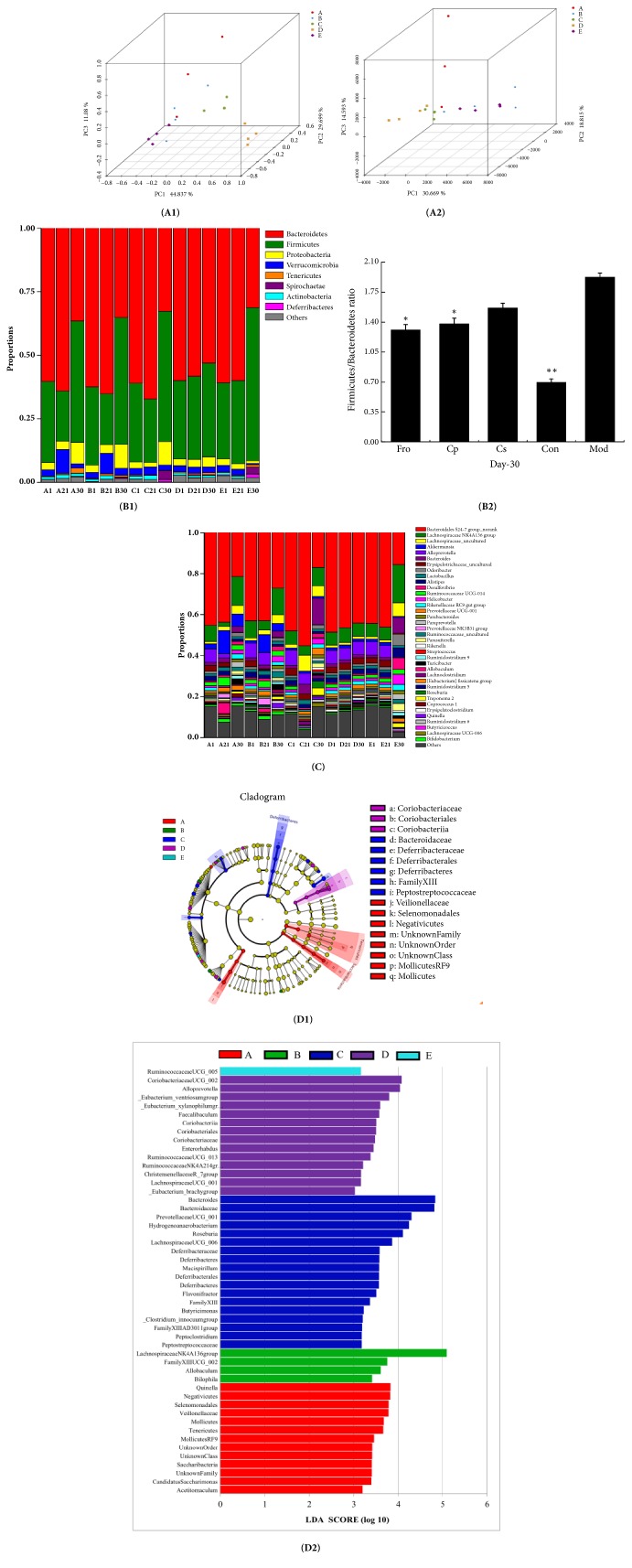
Gut microbial community structure in mice after treatment with* Codonopsis pilosula* extracts. (A1) Multiple sample PCoA. (A2) Multiple sample PCA. (B1) Microbial community bar plot by phylum. (B2)* Firmicutes*/*Bacteroidetes* ratio. (C) Microbial community bar plot by genus. (D1) Distribution histogram based on LDA of differences in dominant microorganisms between groups. (D2) Cladogram. ^*∗*^*P* < 0.05; ^*∗∗*^*P* < 0.01; ^*∗∗∗*^*P* < 0.001 versus the DSS-treated group. Data are presented as means ± SD of four mice in each group (A–D, n = 4). A = fructooligosaccharides group, B =* Codonopsis pilosula* polysaccharide group, C =* Codonopsis pilosula* saponins group, D = control group, and E = DSS-induced colitis group.

Next, we performed sequencing analysis to determine structural changes in the gut microbiota in the five studied groups from days 1, 21, and 30 of the experimental period. In total, 2716932 usable reads and 787 operational taxonomic units (OTCs) were obtained from the 60 samples. Unsupervised multivariate statistical methods, including principle component analysis (PCA) and UniFrac distance-based principle coordinate analysis (PCoA), were used to analyze overall structural changes in the gut microbiota. The results of PCA (Figure 4(A2)) and UniFrac PCoA (Figure 4(A1)) showed that the overall structure of the gut microbiota had changed greatly in mice with DSS-induced colitis. There were significant differences between the Mod and Con groups; however, the drug groups (Fro, Cp, and Cs) showed overall gut microbiota structures similar to those of the Con group. Of all drug groups, the Cs group was the most similar to the Con group.

The gut microbiota community structure reflects the microbial species and their relative abundances. As shown in Figure 4(B1), all groups contained* Bacteroidetes*,* Firmicutes*,* Proteobacteria*,* Verrucomicrobia*, and* Actinobacteria*. At the phylum level, in the prevention period, the abundances of* Bacteroidetes* and* Actinobacteria* in different drug groups were markedly increased (*p* < 0.05) compared with that in the Con group, whereas* Firmicutes* was markedly reduced (*p *< 0.05). In addition, the abundance of* Verrucomicrobia* was markedly increased in the Fro and Cp groups (*p *< 0.05). During modeling, the abundances of* Bacteroidetes*,* Verrucomicrobia*,* Proteobacteria*, and* Actinobacteria* were significantly decreased in the Mod group compared with the control group (*p *< 0.01), whereas the abundances of* Firmicutes*,* Tenericutes*,* Deferribacteres*, and* Spirochaetae* were significantly increased (*p *< 0.01). In addition, the abundances of* Proteobacteria* in the Fro, Cp, and Cs groups were higher than those in the Mod group (*p *< 0.01); Cp and Fro could selectively inhibit the growth of* Spirochaetae* (*p* < 0.01), whereas Cp and Cs could selectively inhibit the growth of* Tenericutes* (*p* < 0.05). Moreover, the abundance ratio of* Firmicutes*/*Bacteroidetes* (F/B) in the Mod group was significantly increased compared with that in the Con group (*p *< 0.01), which was markedly higher than those in the Fro and Cp groups (*p *< 0.05; Figure 4(B2)).

At the genus level, as shown in Figure 4(C),* Bacteroidales* S24−7 group_norank,* Lachnospiraceae* NK4A136 group,* Lachnospiraceae*_uncultured,* Akkermansia*,* Alloprevotella*,* Bacteroides*,* Erysipelotrichaceae*_uncultured,* Odoribacter*, and* Lactobacillus* strains accounted for the majority of microflora in all groups. During the prevention period, the abundances of* Odoribacter*,* Lachnospiraceae* NK4A136 group, and* Rikenellaceae* RC9 gut group in all drug groups were significantly inhibited compared with those in the control group (*p *< 0.05), whereas Cp and Fro selectively promoted the growth of* Akkermansia*,* Lactobacillus*,* Allobaculum*,* Prevotellaceae* NK3B31 group,* Bifidobacterium*, and* Ruminococcaceae* UCG-014 strains (*p *< 0.01). Cp selectively inhibited the growth of* Erysipelotrichaceae*_uncultured compared with that in the Con group (*p *< 0.05), and the abundances of* Lachnospiraceae*_uncultured and* Lachnoclostridium* were markedly increased in the Cs group compared with that in the Con group (*p *< 0.05). After the DSS-induced model period, the abundances of the dominant bacteria, i.e.,* Bacteroidales* S24-7 group_norank,* Alloprevotella*,* Erysipelotrichaceae*_uncultured,* Prevotellaceae* UCG-001, and* Akkermansia*, were significantly reduced in the Mod group compared with those in the Con group (*p *< 0.01), whereas the abundances of* Alistipes*,* Lachnospiraceae* NK4A136 group,* Bacteroides*,* Desulfovibrio*,* Helicobacter*,* Parasutterella*,* Paraprevotella*,* Parabacteroides*, and* Odoribacter* were significantly increased (*p *< 0.01). Following treatment with different drugs, the abundances of* Desulfovibrio*,* Alistipes*,* Helicobacter*,* Parasutterella*, and* Odoribacter* were reduced compared with those in the Mod group (*p *< 0.05). In addition, the abundances of* Alloprevotella*,* Bacteroides*,* Roseburia*, and* Treponema* 2 were selectively increased following treatment with Cs compared with those in the Mod group (*p *< 0.05). Finally, the abundances of* Bifidobacterium*,* Lactobacillus*,* Turicibacter*, and* Quinella* were markedly higher in the Fro and Cp groups than in the Mod group (*p *< 0.05), whereas the abundances of Bacteroides were markedly lower in the Fro and Cp groups compared with that in the Mod group (*p *< 0.05).

The gut microbiota diversity in all groups was then analyzed by linear discriminant analysis (LDA) effect size. A histogram of LDA scores was plotted to identify statistically significant biomarkers and to reveal the dominant microorganisms in each group (Figure 4(D1)). As shown in Figure 4(D2), 13 species were identified as belonging to the phyla Saccharibacteria, Tenericutes, and Firmicutes, which were the most abundant bacteria in the Fro group but not in the other groups. In the Cp group, there were four species belonging to the phyla Proteobacteria and Firmicutes played a key role; 18 species were identified as belonging to the phyla Firmicutes, Deferribacteres, and Bacteroidetes in the Cs group but not in the other groups. In the Con group, 14 species were identified as belonging to the phyla Actinobacteria, Firmicutes, and Bacteroidetes, whereas only the* Ruminococcaceae* UCG_005 genus belonging to Firmicutes was identified in the Mod group. Overall, the diversity of the intestinal flora was reduced in mice with DSS-inducted colitis.

### 3.5. Changes in SCFAs in Feces

**Figure 5:**
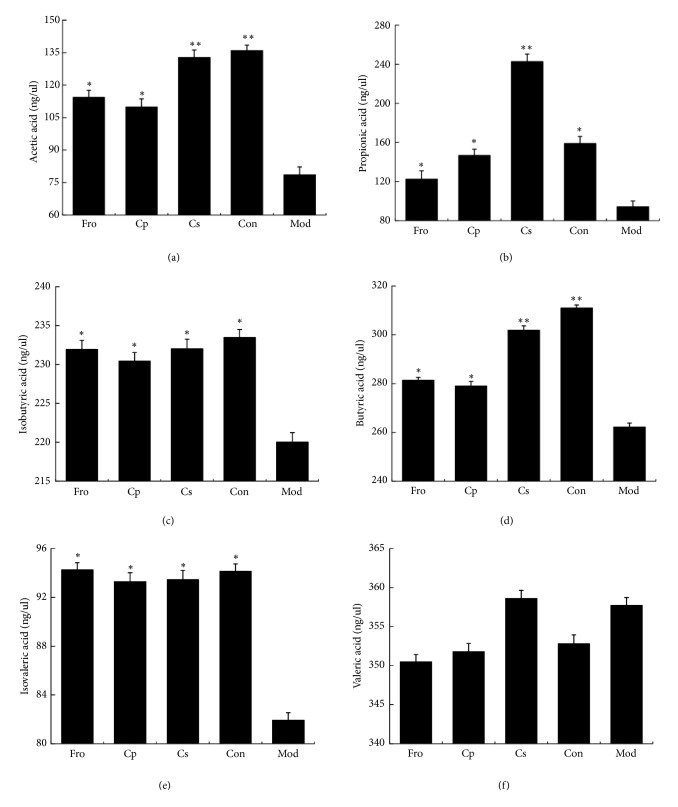
Changes in short-chain fatty acids (SCFAs) in feces after treatment with* Codonopsis pilosula* extracts. (a) The contents of acetic acid, (b) propionic acid, (c) isobutyric acid, (d) butyric acid, (e) isovaleric acid, and (f) valeric acid in each group. ^*∗*^*P* < 0.05; ^*∗∗*^*P* < 0.01; ^*∗∗∗*^*P* < 0.001 versus the DSS-treated group. Data are presented as means ± SD of four mice in each group (a–f, n = 4). Fro = fructooligosaccharides group, Cp =* Codonopsis pilosula* polysaccharide group, Cs =* Codonopsis pilosula* saponins group, Con = control group, and Mod = DSS-induced colitis group.

SCFAs are one of the most important signaling molecules for transferring the physiological and psychological functions of gut microbiota to the host [57]. Changes in the contents of SCFAs after DSS-dependent induction of colitis were studied by GC analysis. As shown in Figures 5(a)–5(f), the contents of acetic acid, propionic acid, butyric acid, isobutyric acid, and isovaleric acid were significantly reduced in mice with DSS-induced colitis compared with those in the Con group (*p *< 0.05). Treatment with different drugs caused increased levels of acetic acid, propionic acid, butyric acid, isobutyric acid, and isovaleric acid (*p *< 0.05), among which the contents of acetic acid, propionic acid, and butyric acid in the Cs group were significantly higher than those in the Mod group (*p *< 0.01). Notably, however, there were no significant differences in the contents of valeric acid in all groups.

### 3.6. Changes in the Abundance of SCFA-Producing Bacteria

**Figure 6:**
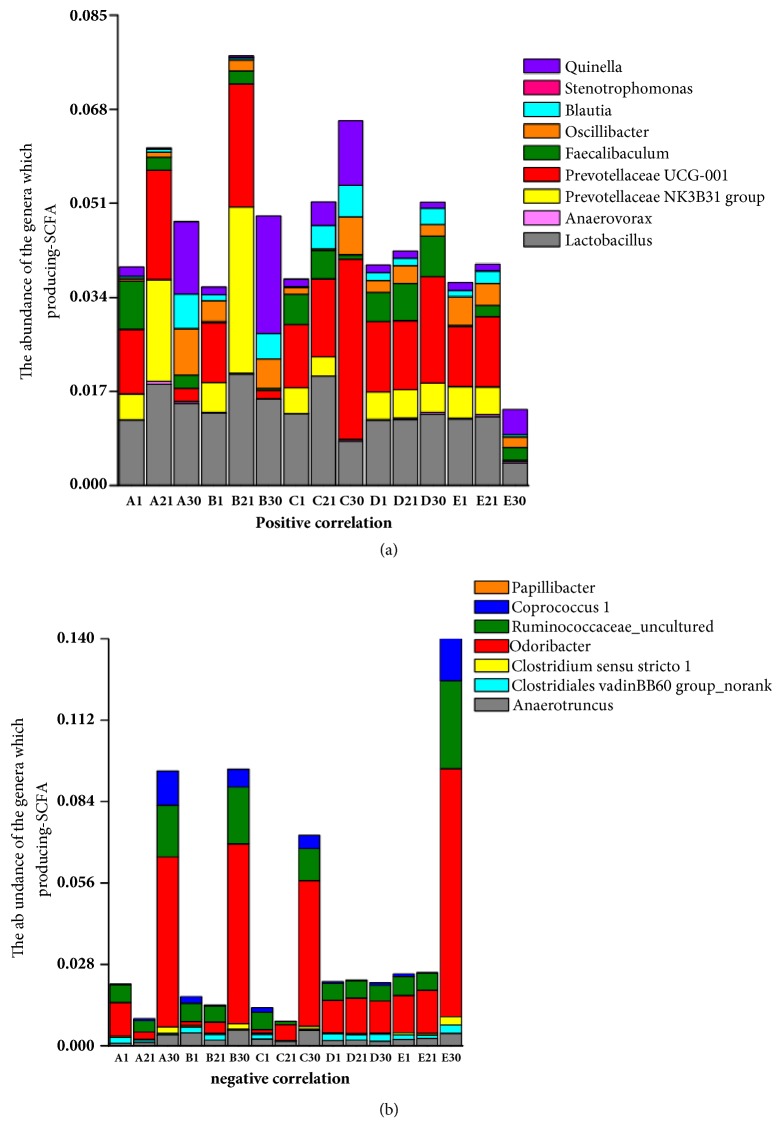
Correlations between the fecal microbiota and fecal SCFA concentrations after treatment with* Codonopsis pilosula* extracts. (a) Positive correlation between the abundance of genera and SCFA concentrations. (b) Negative correlation between the abundance of genera and SCFA concentrations. Fro = fructooligosaccharides group, Cp =* Codonopsis pilosula* polysaccharide group, Cs =* Codonopsis pilosula* saponins group, Con = control group, and Mod = DSS-induced colitis group.

In this study, we examined the relationship between SCFA contents and SCFA-producing bacteria (Figure 6**)**. As shown in Figure 6(a), the total abundance of bacteria positively related to SCFA production in the prevention period was increased after oral administration of different drugs compared with that in the Con group (*p *< 0.05). Specifically, the abundance of SCFA-producing bacteria was the highest in the Cp group (*p *< 0.01). By DSS-dependent induction of colitis, the total abundance of bacteria positively related to SCFA production was significantly reduced in mice with DSS-induced colitis compared with that in the Con group (*p *< 0.01); however, in the different drug groups, the total abundance of SCFA-producing bacteria was markedly higher in drug-treated groups than in the Mod group (*p *< 0.05). In addition, in the Cs group, the abundance of SCFA-producing bacteria showed continuous growth, regardless of the impact of DSS-induced colitis. Specifically, the abundances of* Prevotellaceae* NK3B31 and* Prevotellaceae* UCG-001 in Fro and Cp groups were markedly reduced by induction of colitis (*p *< 0.05), whereas the abundance of* Prevotellaceae* UCG-001 in the Cs group was selectively promoted (*p *< 0.01). In addition, the abundances of* Blautia*,* Oscillibacter*, and* Quinella* were markedly higher in all drug groups than in the Mod group (*p *< 0.05). As shown in Figure 6(b), the total abundance of bacteria negatively related to SCFA production was markedly reduced after oral administration of different drugs compared with that in the Con group during the prevention period (*p *< 0.05). In particular, the abundance in the Cs group was significantly lower than that in the Con group. In mice with DSS-induced colitis, the total abundance of bacteria negatively related to SCFA production was significantly increased compared with that in the Con group (*p *< 0.01); however, in mice treated with different drugs, the total abundance of bacteria negatively related to SCFA production was markedly lower than that in the Mod group (*p *< 0.05). The abundances of* Ruminococcaceae*_uncultured,* Coprococcus* 1,* Odoribacter*, and* Clostridiales* vadinBB60 group_norank were significantly increased by induction of colitis compared with those in the Con group (*p *< 0.01). However, the abundances of* Coprococcus* 1 and* Clostridiales* vadinBB60 group_norank were markedly lower in the Fro, Cp, and Cs groups than in the Mod group (*p *< 0.05). In addition, the Cs group showed weak growth of* Clostridium* sensu stricto 1 in mice with DSS-induced colitis (*p *< 0.01).

### 3.7. Cp Enhanced the Systemic Absorption of Cs in Mice with DSS-Induced Colitis

**Figure 7:**
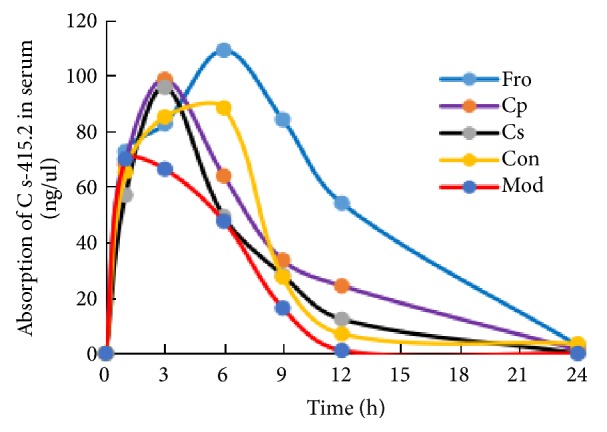
Serum absorption of the largest monomer ion pair (m/z 415.2) after oral administration of Codonopsis pilosula saponins to mice. Fro = fructooligosaccharides group, Cp = Codonopsis pilosula polysaccharide group, Cs = Codonopsis pilosula saponins group, Con = control group, and Mod = DSS-induced colitis group.

The pharmacokinetics of Cs-415.2 after oral administration of Cs extracts were studied by UPLC-TQ-MS. The PKsolver method described above was successfully applied to a pharmacokinetic study in which serum concentrations of Cs-415.2 were determined for 24 h after oral administration (300 mg/kg, n = 3), and the main pharmacokinetic parameters in the Con, Mod, Cp, Fro, and Cs groups are given in Table 2. Compared with the Con group, mice in the Mod group showed changes in the pharmacokinetic behaviors of Cs-415.2. Specifically, the AUC_0-inf_ and C_max_ of Cs-415.2 were reduced markedly in the Mod group compared with those in the Con group (*p *< 0.05). However, the AUC_0-inf_ and C_max_ of Cs-415.2 were improved following treatment with Cp and Cs compared with those in the Mod group (*p* < 0.05). Additionally, the AUC_0-inf_ and C_max_ of Cs-415.2 in the Fro group were significantly increased compared with those in the Mod group (*p* < 0.01).  Figure 7 shows the content change of Cs-415.2 in serum within 24 h. We found that the absorption of serum Cs-415.2 within 24 h was as follows: Fro group > Con group > Cp group > Cs group > Mod group. In addition, the maximum absorption of Cs-415.2 in serum between Fro group and control group was about 6 h; Cp group and Cs group were about 3 h, while the Mod group was about 1 h.

## 4. Discussion

CPN is clinically used for replenishing Qi (vital energy) deficiency, and the importance of the pharmacological activity of CPN has been highlighted based on the wide use of this HM. A water-soluble polysaccharide with a high molecular weight of more than 10 kDa was isolated from the roots of CPN [58, 59]. Furthermore, a pectic polysaccharide with a molecular mass of 14.5 kDa was at first isolated from CPN [60]. These findings imply that bioactive polysaccharides in CPN may cover a wide molecular weight distribution.

Current studies on HM chemicals are limited by a lack of standards, low biological concentrations, and large variation. For example, Cs typically obtained from laboratory self-separation and extraction techniques resulted in low quality and yield [49]. Therefore, in this study, we examined the effects of Cs by using the highest concentration of compounds in Cs extracts, and our results were expected to reflect the metabolism of Cs in mice with DSS-induced colitis. The monomer formula is C_29_H_50_O, which has the same relative molecular mass as Stigmast-7-en-3-ol and its a type of triterpenoid, isolated from the CHCl_3_-soluble fraction of the methanol extract of CPN [47].

In this study, DSS-induced acute colitis mice model was established, consistent with previous individual studies of DSS-induced colitis [50], a well-established model to imitated gut microbial dysbiosis [24]. Using different taxonomic levels of bacterial population analysis, the difference between gut microflora structure and diversity in mice with acute colitis after three drugs intervention was investigated. At the phylum level, the abundance of* Bacteroidetes* and* Actinobacteria* increased by three drugs intervention, whereas Fro and Cp significantly increased the proportion of* Verrucomicrobia*.* Bacteroidetes* encodes 3976 carbohydrate-active enzymes, which are essential for macromolecular metabolism [61]. Although some* Actinobacteria* can cause human diseases, some strains, particularly* Streptomyces *spp., can metabolize certain drugs to produce many biologically active metabolites, thereby playing a role in antibacterial [62], antiviral, and immune regulation [63]. Certain* Verrucomicrobia* are able to metabolize sulfur and degrade mucins [64]. During the DSS-induced model period, the abundance of* Proteobacteria* increased significantly after intervention with three drugs; but Cp and Fro inhibited the growth of* Spirochaetae*. It has been documented that many strains of* Proteobacteria* can induce the secretion of IgA and regulate intestinal homeostasis [65], while most strains of* Spirochaetae* have pathogenicity [66], which may increase the pathological process of colitis. The F/B ratio is not only closely related to obesity, but also a key indicator for evaluating the inflammatory response of IBD. We found that the ratio of F/B in the intestinal tract of mice with acute colitis was significantly increased compared with the control group, which is consistent with the literature report [67].

At the genus level, our laboratory studies have shown that low-purity Cp (35%) increase the proportion of beneficial bacteria in* Bifidobacterium* spp. and* Akkermansia* spp. and reduce the abundance of harmful bacteria in DSS-induced colitis. Additionally, when the purity of Cp was increased 56%, Cp imparted a prebiotic-like effects in mice with DSS-induced colitis by simultaneously stimulating the growth of the two most important probiotics, i.e.,* Bifidobacterium* spp. and* Lactobacillus* spp., inhibiting the abundance of* Desulfovibrio *spp.,* Alistipes *spp., and* Helicobacter *spp*.,* which rectified the disorder of gut microbiota in colitis mice. Its colonic regulation effect was significantly better than Cs group, and there is no significant difference with Fro, which has been recognized as having a probiotic effect.* Alistipes *spp. [68],* Desulfovibrio* spp. [69], and* Helicobacter* spp. [70] may be pathogens bacteria of IBD.* Desulfovibrio *spp. is a typical representative of sulfate-reducing bacteria and produces hydrogen sulfide with damaged epithelial cells by degrading SCFAs and amino acids [69]. At the same time, the results are consistent with previous experiments demonstrating that polysaccharides from HM promote the growth of gut bacteria to exert prebiotic-like effects and selectively stimulate the growth of symbiotic beneficial bacteria (probiotics) [37, 48, 71]. In addition, we found that the abundance of* Bacteroides* spp. was elevated in DSS-induced colitis, consistent with previous studies demonstrating that enterotoxigenic* Bacteroides* are associated with IBD [72]. Cp and Fro can inhibit the growth of* Bacteroides* spp., although the mechanism of action is unclear. Interestingly, Cs could also stimulate the growth of* Bacteroides* spp. in DSS-induced mice, which may be explained by the observation that* Bacteroides* spp. are the dominant bacterial species involved in the metabolism of saponins [73]. Therefore, Cp also simultaneously stimulated the growth of* Akkermansia* spp., which could alleviate symptoms of type 2 diabetes by induction of Tregs (Foxp3+ T cells) [74]. Additionally, Cp and Fro can promote the growth of* Quinella*,* Allobaculum*, and* Turicibacter*, which are related to the production of SCFAs by fermentation [75] and inhibited the abundance of* Parasutterella* and* Paraprevotella*, which were significantly elevated in patients with IBD and colon cancer [76], respectively. However, because of the limited number of digestible enzymes encoded in the human genome, most herbal polysaccharides are indigestible after oral administration until they reach the intestinal tract. In contrast, the gut microbiome encodes thousands of carbohydrate-active enzymes; for example, the phyla* Firmicutes* encode 4119 CAZymes (39.6 per genome) [61], respectively. It can be fermented by the gut microbe into SCFAs after successive degradation [77]. LEfSe result displayed certain genus in* Firmicutes* belong to the key genus in each experimental group, which may be related to DSS-induced colitis. The reason for the dominant phylum of* Deferribacteres* and* Bacteroidetes* in the Cs group may be that some of these genus are caused by important strains involved in metabolism [61]. In short, whether in the prophylactic or colitis treatment period, the effect of Cp in acute colitis mice can be divided into two aspects: on the one hand, after orally administration of Cp to the intestine, Cp was degraded by thousands of carbohydrate-enzymes encoded by the gut microbiome and was broken down into small molecules by the fermentation of intestinal microbes, which were then absorbed by the host to exert pharmacological activity. On the other hand, Cp provided nutrients for the gut microflora and assisted the gut microflora to compete limited space with pathogenic bacteria, by strengthening the body's immune response, promoting the growth of probiotics in the intestinal tract, inhibiting the colonization of pathogenic bacteria, and restoring the intestinal homeostasis.

The SCFAs produced by the gut microbiota include acetic acid, propionic acid, butyric acid, isobutyric acid, isovaleric acid, and valeric acid, which serve as energy sources for colonocytes [78] and can be absorbed across the gut epithelium into circulation or utilized by enterocytes, thereby having effects both systemically and locally [79]. In the present study, we found that Cp improved SCFA production in DSS-induced mice, increased the systemic and local energy supply of SCFAs, and alleviated malnutrition by selectively enriching four putative SCFA-producing bacteria, i.e.,* Blautia*,* Prevotellaceae* UCG-001,* Oscillibacter*, and* Quinella*, and inhibiting certain competitive bacteria (*Coprococcus* 1,* Odoribacter*, and* Clostridiales* vadinBB60 group_norank) in the gut commensal.* Coprococcus* 1 inhibits the production of propionic acid, and* Odoribacter* is negatively correlated with the production of acetic acid and propionic acid, whereas* Clostridiales* vadinBB60 group_norank inhibits propionic acid and butyric acid [75]. However, in addition to fermentation of Cp, stimulation of SCFA production by gut microbiota also involves the inhibition of histone deacetylases (HDACs) and G-protein-coupled receptors (GPCRs), which are major mechanisms involved in SCFA signaling. SCFA-induced HDAC inhibition decreases proinflammatory cytokine (IL-6, IL-22, etc.) production and deactivates proinflammatory transcription factors (e.g., nuclear factor-*κ*B) in a variety of immune cells, such as Tregs, which largely resulted in anti-inflammatory immune phenotypes [80]. In contrast, GPCRs sense SCFAs and then trigger an array of downstream consequences, such as regulation of colonic Treg homeostasis [40]. In this study, we found that Cp indirectly reduced damage to the colonic epithelial mucosa and restored the Th17/Treg balance in mice with UC by inhibiting the expression of proinflammatory cytokines and promoting the expression of anti-inflammatory cytokines. This may be explained by the effects of polysaccharides on the population of bacteria involved in SCFA metabolism.

In this study, the relative exposure method was applied to analyze the pharmacokinetics of Cs in DSS-induced mice; this method does not depend on standard, relatively high exposure levels in samples of exogenous pharmaceutical ingredients used in the dilution ratio standard curve method (i.e., the standard curve of the monomer compound is obtained by linear regression of the sample with the internal standard peak area ratio) to obtain the relative quantity of monomer compounds. The measured blood drug concentration was not the actual blood concentration of the monomeric compound, but the “relative blood concentration”. This method was compared with pharmacokinetic data obtained by standard tests; and in addition to the absolute blood drug concentrations, the pharmacokinetic parameters were not significantly different [81]. Thus, this method has enabled us to solve some technical issues associated with current pharmacological analysis of HMs. In this study, Cs was orally administered (300 mg/kg) to DSS-induced mice once a day, and the absorption rate and effect time of Cs in the serum were markedly enhanced by Cp-dependent restoration of gut microbial dysbiosis; thus, Cp improved the therapeutic effect of Cs in DSS-induced mice due to the metabolism of saponins by* Lactobacillus* spp. and* Bifidobacterium* spp. [38]. In addition, the relative abundances of* Lactobacillus* spp. and* Bifidobacterium* spp. in the model mice were decreased in this study. Given their above-described roles, such variations were expected to weaken intestinal metabolism and absorption of Cs. Interestingly, we found that when Cs was administered separately for a period of time, although the regulatory effects of Cs on gut microbiota were weaker than those of Cp, the absorption of Cs was also markedly enhanced in DSS-induced colitis. This could be attributed to metabolites of microbiota-driven Cs conversion, which would in turn ameliorate changes to the gut microbiota, such as the metabolism of saponins by* Bacteroides* spp. [38]. Moreover, due to their low molecular weight, saponins are more easily absorbed by the body. Certain bacteria belonging to* Akkermansia *[82],* Bifidobacterium *[83], and* Bacteroides *[30] are able to produce SCFAs, which then regulate the homeostasis of Tregs [84] and promote the production of intestinal IgA [85]. The absorption of Cs in DSS-induced mice may be a result of the combine defects of the gut microbiota, immunity, SCFAs, and CPN extract.

## 5. Conclusion

In summary, combining “multiple components against multiple targets” is continually proposed as the therapeutic principle of HM. Our current findings provided a novel IBD model for studying HM metabolism by gut microbiota mediated, in which polysaccharides synergistically functioned with small molecules (saponins) present in the HM decoction. On the one hand, when Cp was used to assist in the limited competition of gut microbiota and pathogens, abnormal Th17/Tregs were gradually balanced by promoting the expression of anti-inflammatory cytokines and inhibiting the secretion of proinflammatory cytokines, which enhanced the body's immune response, inhibited the colonization of pathogenic bacteria, and promoted the pharmacological activity of Cp in anti-inflammatory, antibacterial, and intestinal mucosal integrity eventually. On the other hand, Cp not only provided nutrients for the gut microbiota, but also restored intestinal homeostasis in mice with colitis by upregulating beneficial bacteria in the gut and downregulating the abundance of harmful bacteria, and the recovered gut microbiota utilized metabolic enzymes encoded by its gut microbiome to further promote the decomposition of Cp into small molecule polysaccharides, ultimately promoted the production of SCFAs, and provided sufficient energy for the colon. In this way, a virtuous circle was formed and colitis was relieved. The results revealed a relationship in which polysaccharides, although indigestible by the host directly, potentially acted as prebiotics, leading to favorable changes in the gut microbiota by promoting the growth of probiotics. The improved gut microbiota then enhanced the absorption of saponins coadministered in the CPN. Thus, Cp may be developed as probiotic products to regulate the gut microbiota and can be applied to the synergistic metabolism of HM small molecules in patients with IBD in the future. According to the dose of Cp used in the Chinese Pharmacopoeia, it is suggested that different doses of 9-30g can be selected according to the patient's disease pathological process, but the maximum daily dose can only be 30g. The specific administration time can be based on the patient's relief determined, which can also be determined based on the consumption of fructooligosaccharides. In addition, phytochemical investigations and the widely known bioactive properties of CPN, particularly with regard to the mechanisms through which Cp and Cs are used in ethnomedicine and to support development of pharmaceutical products, will undoubtedly be critical to more advanced research.

## Figures and Tables

**Table 1:** Total mass spectrometry parameters for *C. pilosula* saponins.

Parameter	Mother ion (*m/z*)	Ion (*m/z*)	Collision energy (eV)	Source rupture voltage (eV)
Cs	415.2	415.2	0	105
	415.2	119.1	15	105

Cs: *Codonopsis pilosula* saponins.

**Table 2:** Pharmacokinetic parameters of the largest monomer ion pair (*m/z* 415.2) after oral administration of *C. pilosula* saponins to mice.

	Fro	Cp	Cs	Con	Mod
Parameter			Oral administration (n = 3)		
T_max_ (h)	6	3	3	6	1
C_max_ (ppb)	109 ± 22.9^a^	98.5 ± 14.4^b^	95.7 ± 10.1^b^	88.3 ± 20.4^b^	69.83 ± 10.7^c^
t_1/2_ (h)	3.53 ± 0.33	4.94 ± 0.44	3.85 ± 0.62	3.49 ± 0.38	3.67 ± 0.5
AUC_0-t_ (ppb h)	884.5 ± 65.5	615.55 ± 60	575.55 ± 78	695.45 ± 64.5	538.56 ± 49.9
AUC_0-t_ (ppb h)	898.77 ± 65.9^a^	788.43 ± 59.8^b^	748.43 ± 63.3^b^	761.74 ± 64.4^b^	596.46 ± 50.5^c^
AUMC_0-inf_ (ppb h^2^)	5725.48 ± 535	6133.39 ± 478	5473.32 ± 298	6532.63 ± 567	3636.69 ± 357
MRT_0-inf_ (h)	6.37 ± 1.5	7.79 ± 1	6.79 ± 0.8	8.58 ± 1.2	6.10 ± 0.8
Vz/F (L/h kg)	1.70 ± 0.25	2.13 ± 0.24	2.37 ± 0.18	1.46 ± 0.24	2.66 ± 0.37
Cl/F (L/h kg)	0.33 ± 0.06	0.38 ± 0.1	0.41 ± 0.07	0.39 ± 0.05	0.50 ± 0.14

^a–c^ Data within a column without the same superscripts differ significantly (*P* < 0.05).

Fro = fructooligosaccharides group, Cp = *Codonopsis pilosula* polysaccharide group, Cs = *Codonopsis pilosula* saponins group, Con = control group, and Mod = DSS-induced colitis group.

## Data Availability

All relevant data have been reflected in the article.
